# 15-Hydroxyprostaglandin Dehydrogenase Inhibitor Restores Endothelial Function Under Dihydrotestosterone-Induced Stress in Human Dermal Microvascular Endothelial Cells

**DOI:** 10.3390/molecules31010123

**Published:** 2025-12-29

**Authors:** Mujun Kim, Hak Joong Kim, Yurim Lee, Sanghwa Lee, Dong Chul Lim, Hee Dong Park, Dong Wook Shin

**Affiliations:** 1 Research Institute for Biomedical and Health Science, Konkuk University, Chungju 27478, Republic of Korea; besy100@kku.ac.kr; 2 Innovo Therapeutics Inc., 507, Mapo-daero 38, Mapo-gu, Seoul 04174, Republic of Korea; hakkim@innovothera.com (H.J.K.); yrlee@innovothera.com (Y.L.); shlee@innovothera.com (S.L.); dclim@innovothera.com (D.C.L.); hdpark@innovothera.com (H.D.P.)

**Keywords:** dihydrotestosterone, endothelial function, human dermal microvascular endothelial cells, hair growth, 15-prostaglandin dehydrogenase inhibitor

## Abstract

Androgenetic alopecia (AGA) is closely associated with oxidative stress and vascular dysfunction, which disrupt nutrient delivery to hair follicles and promote follicle miniaturization. Dihydrotestosterone (DHT) exposure impairs human dermal microvascular endothelial cell (HDMEC) function by inducing mitochondrial disruption, excessive reactive oxygen species (ROS) accumulation, and reduced angiogenic capacity. This study evaluated the protective effects of dihydroisoquinolinone piperidinylcarboxy pyrazolopyridine (DPP), a novel 15-hydroxyprostaglandin dehydrogenase (15-PGDH) inhibitor identified through the AI-based discovery platform DeepZema^®^, in DHT-exposed HDMECs. DPP markedly reduced intracellular and mitochondrial ROS levels, restored mitochondrial membrane potential, and increased ATP production, thereby alleviating oxidative stress and supporting mitochondrial function. DPP also enhanced endothelial cell migration and capillary-like tube formation, demonstrating the restoration of angiogenic capacity that is essential for sustaining perifollicular vascularization. Moreover, DPP mitigated stress-associated signaling by reducing the phosphorylation of ERK, JNK, and p38 within the MAPK pathway, thereby suggesting the reestablishment of endothelial homeostasis under DHT-induced stress. Collectively, these findings indicate that DPP preserves endothelial function under DHT-driven oxidative conditions. We suggest that DPP may exert complementary protective effects on both vascular and follicular compartments, supporting its potential relevance in hair follicle regeneration.

## 1. Introduction

Androgenetic alopecia (AGA) represents the most prevalent type of hair loss in men [1,2]. It is characterized by progressive thinning of hair in androgen-sensitive areas, such as the frontal, temporal, and vertex regions of the scalp, due to the miniaturization of hair follicles [3,4,5]. The condition is driven by genetic susceptibility and androgen activity, particularly dihydrotestosterone (DHT) [6]. DHT, derived from testosterone, binds to androgen receptors in hair follicles and initiates processes that shorten the anagen phase and lead to follicle miniaturization [7,8,9]. This process is further exacerbated by increased 5α-reductase activity, the enzyme that converts testosterone to DHT within hair follicles [10,11]. Current pharmacological treatments for AGA include 5α-reductase inhibitors such as finasteride and topical vasodilators such as minoxidil (MIX) [12]. MIX enhances perifollicular blood flow but may cause local irritation and unwanted hair growth [13]. Finasteride reduces DHT levels but can lead to systemic adverse effects, including decreased libido and erectile dysfunction [14]. These limitations underscore the need for alternative or complementary therapeutic strategies that target additional biological pathways involved in follicular and vascular maintenance.

Hair follicle regeneration is closely regulated by prostaglandins, which serve as key mediators of tissue homeostasis and repair [15,16]. Among these, prostaglandin E2 (PGE_2_) has been reported to activate stem cells and promote tissue regeneration [17]. Similarly, prostaglandin F_2α_ (PGF_2α_) analogs, such as bimatoprost, have received FDA approval for enhancing eyelash growth and have demonstrated stimulatory effects on cultured human hair follicles [18,19,20,21,22]. These findings highlight the therapeutic potential of targeting prostaglandin pathways in the treatment of hair loss. 15-PGDH, the key enzyme responsible for prostaglandin degradation, modulates the local availability of PGE_2_. Inhibition of this enzyme has been proposed to enhance tissue regeneration, improve vascular function, and potentially promote hair follicle activity through increased prostaglandin signaling [23,24]. To further investigate this approach, we applied DeepZema^®^, an AI-based drug discovery platform, and identified dihydroisoquinolinone piperidinylcarboxy pyrazolopyridine (DPP) as a novel inhibitor of 15-hydroxyprostaglandin dehydrogenase (15-PGDH), the key enzyme responsible for prostaglandin degradation. Our previous work demonstrated that DPP protected human follicle dermal papilla cells (HFDPCs) from DHT-induced damage, thereby supporting its therapeutic potential for hair growth [25].

In addition to dermal papilla cells, the perifollicular microvascular network plays a pivotal role in hair growth by delivering oxygen and nutrients essential for follicular activity [26,27]. The deterioration of this vasculature has been associated with hair loss, whereas dynamic vascular remodeling is critical for maintaining the anagen phase of the hair cycle [28,29]. Recent studies have shown that a balding scalp exhibits microvascular abnormalities and regression of capillaries surrounding the dermal papilla [30]. These vascular alterations suggest that androgen signaling plays a key role in perifollicular vascular remodeling [31]. Therefore, we used DHT to induce endothelial stress in HDMECs to model androgen-related changes in the hair follicle microvasculature. The clinical relevance of vascular regulation in hair biology is further highlighted by MIX, an FDA-approved antihypertensive vasodilator that was later repurposed as a hair-growth agent [32]. By increasing perifollicular blood flow and enhancing the microenvironmental support of dermal papilla cells, MIX demonstrates how vascular protection and the reduction in oxidative stress can indirectly improve follicular function [33,34,35,36]. Collectively, these findings highlight the interconnected roles of microvascular stability, redox homeostasis, and follicular biology in maintaining hair growth.

Human dermal microvascular endothelial cells (HDMECs) form capillary networks in proximity to dermal papilla cells [37,38]. These endothelial cells are not only responsible for maintaining local blood supply but also actively interact with dermal papilla cells through paracrine signaling and angiogenic mediators, thereby sustaining the regenerative capacity of hair follicles [39,40,41].

In this study, we investigated the potential role of DPP by evaluating its protective effects on DHT-damaged HDMECs, which are essential for maintaining the microvascular environment surrounding hair follicles.

## 2. Results

### 2.1. Effects of DPP on HDMECs Viability and Proliferation

**Figure 1:**
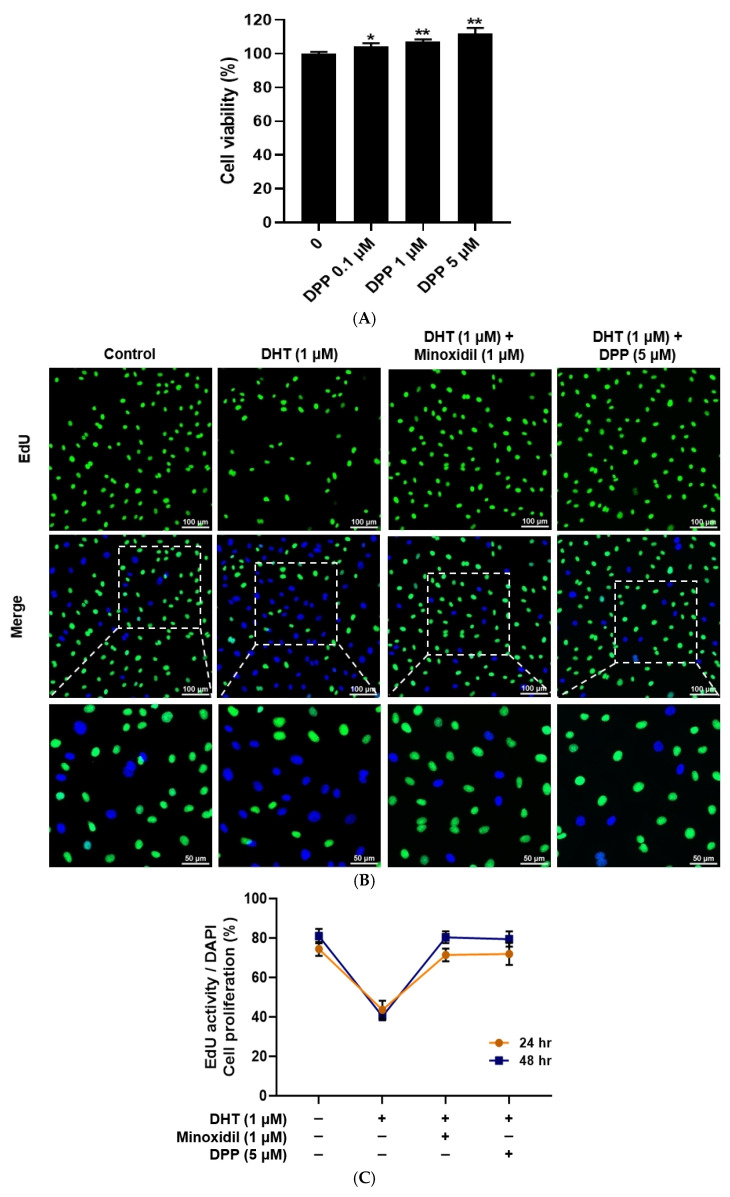
Effects of DPP on cell viability and proliferation in HDMECs. (**A**) Cell viability of DPP at different concentrations was assessed using the MTT assay. (**B**) Representative EdU fluorescence images showing proliferative activity in HDMECs treated with 1 µM DHT followed by 5 µM DPP or 1 µM MIX for 48 h. Proliferating cells were identified by EdU incorporation (green), while total nuclear DNA was visualized by DAPI staining (blue) (scale bar, 100 µm). (**C**) Cell proliferation was further evaluated after 24 and 48 h of DPP treatment using the EdU staining assay. Cell viability and proliferation were expressed as the percentage (%) relative to the untreated control group. Data are presented as mean ± SD (n = 3) based on three independent experiments. * *p* < 0.05 and ** *p* < 0.01 compared with the control group.

The cytotoxicity of DPP in HDMECs was evaluated using an MTT assay. DPP treatment at concentrations of 0.1, 1, and 5 µM did not induce any detectable cytotoxicity. Moreover, DPP increased the viability of HDMECs compared with the control (Figure 1A). Under these experimental conditions, DHT treatment reduced cell proliferation, whereas DPP increased the number of EdU-positive cells compared with the DHT-treated group (Figure 1B,C).

### 2.2. DPP Promoted the Migration of DHT-Damaged HDMECs

**Figure 2:**
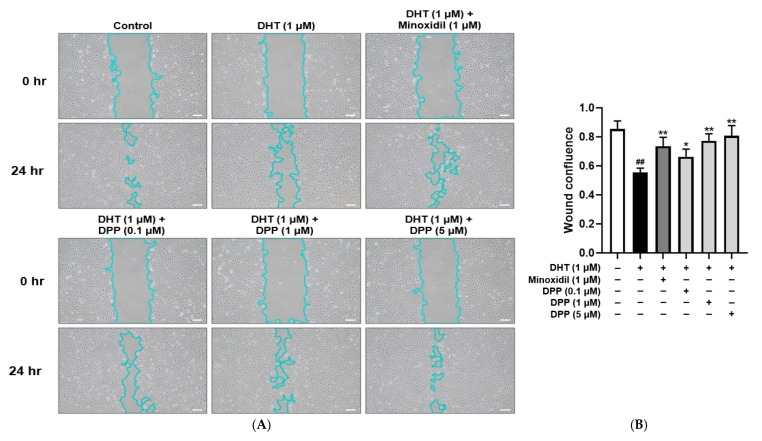
Wound healing effects of DPP on HDMECs stimulated with 1 µM DHT. Cells were treated with DPP (0.1, 1, and 5 µM) or 1 µM MIX for 24 h. (**A**) Representative phase-contrast images showing wound closure in HDMECs at 0 and 24 h (scale bar, 20 µm). Cell outlines are shown in light blue. Images represent results from three independent experiments. (**B**) Quantitative analysis of wound closure area measured using ImageJ software (version 1.53e). Data are presented as mean ± SD (n = 3), with statistical significance denoted as * *p* < 0.05 and ** *p* < 0.01 relative to the DHT-treated group. ## *p* < 0.01 compared with the control group.

The migration of HDMECs is essential for hair follicle vascularization and regeneration [42]. To investigate the effects of DPP on the migratory capacity of DHT-damaged HDMECs, a wound healing assay was performed. Cells treated with DHT alone showed significantly delayed wound closure compared with the control, indicating that DHT impaired cell migration. In contrast, treatment with DPP or MIX markedly enhanced wound closure after 24 h relative to DHT treatment alone (Figure 2).

### 2.3. DPP Suppressed ROS Levels in DHT-Damaged HDMECs

**Figure 3:**
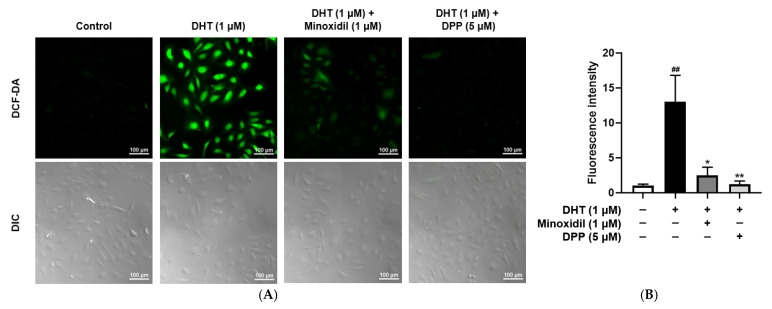
Effects of DPP on ROS levels in HDMECs stimulated with 1 µM DHT. Cells were treated with 1 µM DHT followed by 5 µM DPP or 1 µM MIX for 24 h. (**A**) Representative DCF-DA fluorescence images showing intracellular ROS accumulation (green fluorescence). Images represent results from three independent experiments. (scale bar, 100 µm). (**B**) Quantitative analysis of relative fluorescence intensity was performed using ImageJ software (version 1.53e). Data are presented as mean ± SD (n = 3), with statistical significance denoted as * *p* < 0.05 and ** *p* < 0.01 relative to the DHT-treated group. ## *p* < 0.01 compared with the control group.

Elevated ROS levels are known to impair vascular function by disrupting endothelial homeostasis and compromising structural integrity [43]. To evaluate the ability of DPP to reduce ROS, intracellular ROS levels were measured using DCF-DA staining. As expected, DHT-treated HDMECs exhibited significantly higher ROS levels compared to the untreated control group. Treatment with MIX markedly reduced ROS accumulation in DHT-exposed cells. Similarly, DPP treatment effectively suppressed DHT-induced ROS elevation, restoring ROS levels to levels comparable to the control (Figure 3).

### 2.4. DPP Restored Mitochondrial Membrane Potential in DHT-Damaged HDMECs

**Figure 4:**
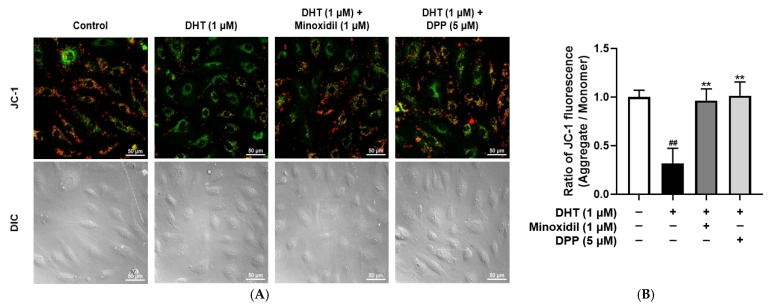
Effects of DPP on mitochondrial membrane potential in HDMECs stimulated with 1 µM DHT. Cells were treated with 1 µM DHT followed by 5 µM DPP or 1 µM MIX for 24 h, and mitochondrial membrane potential was assessed using the JC-1 assay. (**A**) Representative JC-1 fluorescence images showing green fluorescence representing depolarized mitochondria and red fluorescence indicating hyperpolarized mitochondria (scale bar, 50 µm). Images represent results from three independent experiments. (**B**) Mitochondrial membrane potential was quantified using ImageJ software (version 1.53e). Data are presented as mean ± SD (n = 3), with statistical significance denoted as ** *p* < 0.01 relative to the DHT-treated group. ## *p* < 0.01 compared with the control group.

Mitochondria play a critical role in sustaining cellular energy demands, particularly during the highly proliferative anagen phase of the hair cycle [44,45]. To examine the effects of DPP on the membrane potential of mitochondria in DHT-damaged HDMECs, a JC-1 assay was performed. In this assay, red fluorescence indicates polarized, functional mitochondria, whereas green fluorescence reflects depolarized, dysfunctional mitochondria. Exposure to DHT markedly increased green fluorescence intensity compared with the control group, indicating mitochondrial impairment. By contrast, DPP treatment enhanced red fluorescence (Figure 4).

### 2.5. DPP Reduced Mitochondrial ROS Levels in DHT-Damaged HDMECs

**Figure 5:**
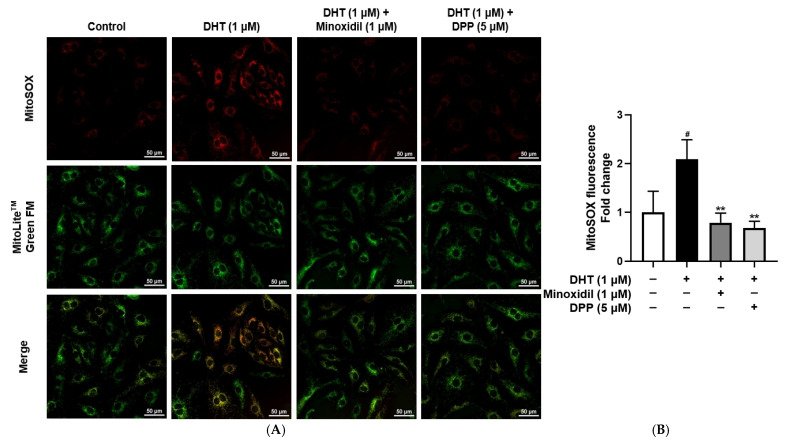
Effects of DPP on mitochondrial ROS levels in HDMECs stimulated with 1 µM DHT. Cells were treated with 1 µM DHT followed by 5 µM DPP or 1 µM MIX for 24 h, and mitochondrial ROS levels were evaluated using fluorescence staining. (**A**) Representative fluorescence images showing red fluorescence indicating mitochondrial ROS and green fluorescence representing mitochondria (scale bar, 50 µm). Images represent results from three independent experiments. (**B**) Quantitative analysis of mitochondrial ROS fluorescence intensity was performed using ImageJ software (version 1.53e). Data are presented as mean ± SD (n = 3), with statistical significance denoted as ** *p* < 0.01 relative to the DHT-treated group. # *p* < 0.05 compared with the control group.

Mitochondria are the primary source of intracellular ROS, and excessive mitochondrial ROS production is a hallmark of mitochondrial dysfunction [46,47]. To specifically assess mitochondrial ROS, a MitoSOX™ Red assay was performed. As expected, DHT exposure markedly increased mitochondrial ROS levels in HDMECs compared with the untreated control group, consistent with mitochondrial impairment. In contrast, treatment with DPP significantly reduced DHT-induced mitochondrial ROS accumulation, restoring levels toward those of control (Figure 5).

### 2.6. DPP Enhanced ATP Production in DHT-Damaged HDMECs

**Figure 6:**
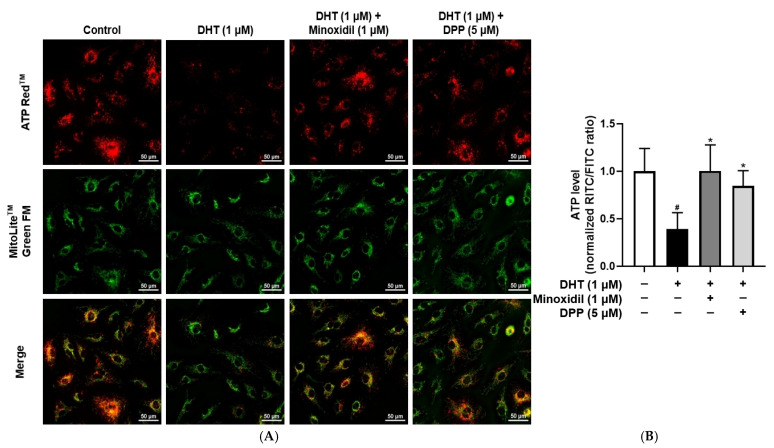
Effects of DPP on ATP levels in HDMECs stimulated with 1 µM DHT. Cells were treated with 1 µM DHT followed by 5 µM DPP or 1 µM MIX for 24 h, and ATP levels were assessed by ATP assay. (**A**) Representative fluorescence images showing red fluorescence representing ATP levels and green fluorescence indicating mitochondria (scale bar, 50 µm). Images represent results from three independent experiments. (**B**) ATP levels were quantified using ImageJ software (version 1.53e), and data are presented as mean ± SD (n = 3). Statistical significance is denoted as * *p* < 0.05 relative to the DHT-treated group. # *p* < 0.05 compared with the control group.

Mitochondria are critical for endothelial cell function by regulating ATP production and maintaining vascular homeostasis [48,49]. To evaluate the effect of DPP on ATP production, intracellular ATP levels were examined using a fluorescence-based detection. In this assay, green fluorescence was used to visualize mitochondrial structures, while red fluorescence represented intracellular ATP levels. Exposure of HDMECs to DHT markedly reduced red fluorescence intensity compared with the control group, indicating impaired ATP production. In contrast, DPP treatment restored red fluorescence signals, reflecting the restoration of mitochondrial ATP synthesis (Figure 6).

### 2.7. DPP Downregulated the Phosphorylation Levels of ERK, JNK, and p38 in DHT-Damaged HDMECs

**Figure 7:**
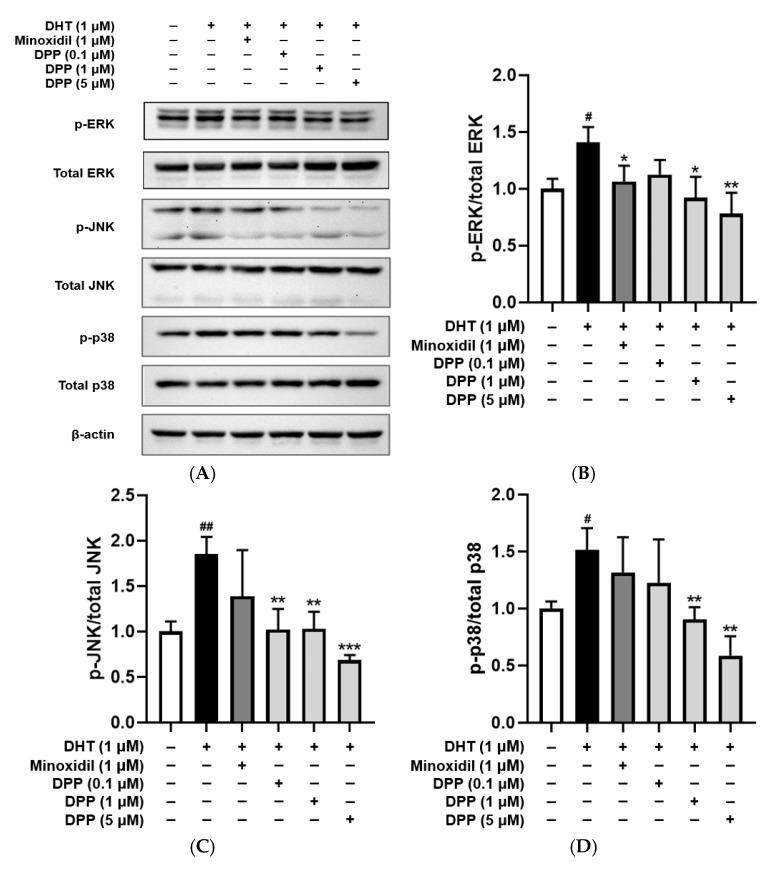
Effects of DPP on the phosphorylation levels of ERK, JNK, and p38 in HDMECs stimulated with 1 µM DHT. (**A**) Representative Western blot images showing the relative expression levels of each protein. (**B**) ERK, (**C**) JNK, and (**D**) p38 relative expression bar graphs. Cells were treated with 1 µM DHT followed by DPP (0.1, 1, and 5 µM) or 1 µM MIX for 24 h. Data are presented as mean ± SD (n = 3), with statistical significance denoted as * *p* < 0.05, ** *p* < 0.01, and *** *p* < 0.001 relative to the DHT-treated group. # *p* < 0.05, and ## *p* < 0.01 compared with the control group.

Dysregulated MAPK activation in endothelial cells has been linked to vascular dysfunction, resulting in impaired angiogenesis and tissue regeneration [50]. The phosphorylation levels of ERK, JNK, and p38 were examined in HDMECs treated with DHT. DHT treatment significantly increased the phosphorylation of ERK, JNK, and p38 compared with the control group. In contrast, treatment with DPP reduced the phosphorylation of these MAPKs (Figure 7).

### 2.8. DPP Improved Tube Formation in DHT-Damaged HDMECs

**Figure 8:**
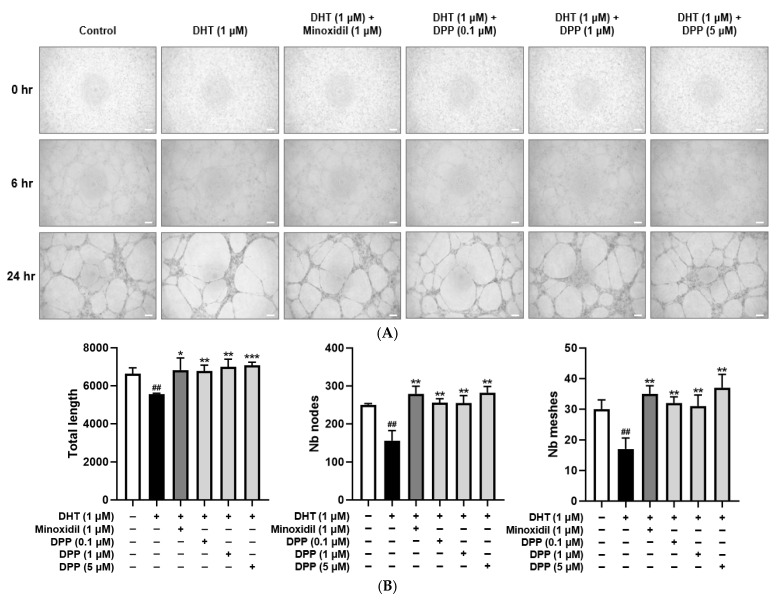
Effects of DPP on tube formation in HDMECs stimulated with 1 µM DHT. Cells were treated with 1 µM DHT followed by DPP (0.1, 1, and 5 µM) or 1 µM MIX for 24 h. (**A**) Representative phase-contrast images showing tube formation in HDMECs at 6 and 24 h (scale bar, 20 µm). Images represent results from three independent experiments. (**B**) Quantitative analysis of tube formation parameters, including total tube length, number of nodes, and meshes, was performed using ImageJ software, version 1.53e. Data are presented as mean ± SD (n = 3), with statistical significance denoted as * *p* < 0.05, ** *p* < 0.01, and *** *p* < 0.001 relative to the DHT-treated group. ## *p* < 0.01 compared with the control group.

A tube formation assay was performed to assess angiogenic activity in HDMECs. Treatment with DHT significantly decreased tube length, mesh formation, and the number of nodes compared with the control group, indicating impaired angiogenic capacity. To examine the protective effect of DPP, HDMECs were co-treated with DHT and DPP to assess the effects of DPP. DPP treatment significantly increased tube length, mesh formation, and the number of nodes relative to the DHT-treated group (Figure 8).

## 3. Discussion

Prostaglandin pathways have been implicated in tissue regeneration and vascular health, suggesting their potential as therapeutic targets for hair loss [51,52,53,54]. Here, we examined the protective effects of DPP, a novel 15-PGDH inhibitor identified through the AI-based discovery platform DeepZema^®^, against DHT-induced endothelial dysfunction.

ROS and mitochondrial dysfunction are widely recognized as critical determinants of hair follicle health and are implicated in the pathogenesis of various forms of hair loss [55,56,57]. Excessive ROS accumulation leads to oxidative damage and impairs the function of HDMECs [58,59]. Mitochondria are essential for supplying cellular energy, and their importance increases during the anagen phase, when cells require large amounts of ATP for growth and metabolism [60]. When mitochondrial function is compromised, ATP levels decline and ROS production increases, leading to a cycle of oxidative stress and cellular injury [61,62,63]. Our results showed that DHT exposure in HDMECs elevated ROS levels, disrupted mitochondrial membrane potential, and impaired ATP production, thereby indicating profound mitochondrial dysfunction. Notably, DPP treatment effectively suppressed both intracellular and mitochondrial ROS, restored mitochondrial membrane potential, and enhanced ATP production. These findings suggest that DPP protects against androgen-induced mitochondrial damage and restores energy homeostasis, thereby counteracting one of the major drivers of endothelial dysfunction and impaired follicular regeneration.

Endothelial cell migration and angiogenesis are fundamental processes for vascular repair and tissue regeneration, as they enable endothelial cells to migrate to injury sites and form new vascular networks [64]. DHT exposure significantly impaired these functions in HDMECs, as evidenced by reduced wound closure and diminished tube formation on Matrigel. DPP treatment reversed these defects by enhancing cell migration and promoting the formation of capillary-like networks, underscoring its ability to restore angiogenic potential under androgen-induced stress. Since endothelial tube formation is closely linked to pro-angiogenic signaling, it is widely recognized that enhanced angiogenic activity is typically associated with the upregulation of growth factors such as VEGF [65,66]. Although angiogenesis contributes to maintaining perifollicular circulation, our findings should be interpreted solely as improvements in endothelial function rather than direct evidence of hair follicle regeneration.

Excessive activation of MAPK signaling in endothelial cells has been implicated in oxidative stress, inflammation, and vascular dysfunction [67,68]. DPP also modulated intracellular signaling associated with endothelial dysfunction. Specifically, it significantly reduced the phosphorylation of ERK, JNK, and p38 in DHT-exposed HDMECs, thereby attenuating MAPK pathway activation. Persistent MAPK phosphorylation is closely linked to oxidative stress, inflammatory signaling, and vascular dysfunction. By inhibiting this pathway, DPP mitigated stress-induced endothelial injury and contributed to the stabilization of the vascular microenvironment.

**Figure 9:**
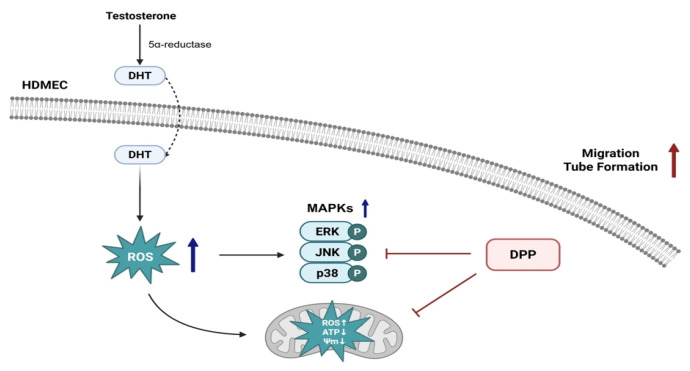
Schematic illustration of the protective role of DPP in HDMECs. DHT, converted from testosterone by 5α-reductase, exerts detrimental effects on endothelial cells. DPP reduces ROS levels, restores damaged mitochondrial function, inhibits MAPK activation, and enhances angiogenic responses such as migration and tube formation in HDMECs, thereby maintaining the perifollicular vasculature essential for hair follicle regeneration.

Taken together, our findings indicate that DPP protects endothelial function and angiogenesis under DHT-induced stress by inhibiting 15-PGDH, thereby supporting the perifollicular vasculature required for hair follicle regeneration (Figure 9). Importantly, our previous study demonstrated that DPP enhances key functional features of HFDPCs, including alkaline phosphatase activity, Wnt/β-catenin signaling, and three-dimensional spheroid formation, which are essential for their hair-inductive capacity [25]. Thus, our findings suggest that DPP may modulate both the vascular and follicular compartments through complementary mechanisms involving protection against oxidative stress and support of critical regenerative pathways. While these combined observations offer a broader conceptual framework linking vascular stability with follicular health, direct in vivo or follicle-specific validation is still required. Therefore, the current findings provide an initial basis for further investigation, and additional preclinical and clinical studies will be necessary to determine whether DPP contributes to hair follicle regeneration.

## 4. Materials and Methods

### 4.1. Cell Culture

HDMECs (Promo Cell, Heidelberg, Germany) were cultivated at 37 °C in a 5% CO_2_ incubator using endothelial cell growth medium (Promo Cell, Heidelberg, Germany) supplemented with 1% penicillin-streptomycin (Welgene Inc., Gyeongsan, Republic of Korea).

### 4.2. Cell Viability Assay

HDMECs were treated with DPP at final concentrations of 0.1, 1, and 5 µM for 24 h. Following incubation, the medium was removed and replaced with medium containing diluted EZ-Cytox solution (DoGenBio, Seoul, Republic of Korea). Cells were then incubated for an additional 1 h, after which absorbance was recorded at 450 nm.

### 4.3. Cell Proliferation Assay

HDMECs were plated in confocal culture dishes and maintained for 24 h. After incubation, the cells were treated with 1 µM DHT (Sigma-Aldrich, St. Louis, MO, USA), 1 µM minoxidil (Sigma-Aldrich), or 5 µM DPP in the presence of 10 µM EdU working solution, and further incubated for 24 h under identical conditions. Following treatment, 1 mL of 3.7% formaldehyde was added to each dish and incubated for 15 min to fix the cells. The samples were rinsed twice with 3% bovine serum albumin (BSA) prepared in Dulbecco’s phosphate-buffered saline (DPBS) (Welgene Inc., Gyeongsan, Republic of Korea), followed by permeabilization in 1 mL of DPBS supplemented with 0.5% Triton X-100 for 20 min. Afterward, the cells were washed twice again with 3% BSA in DPBS, and 0.5 mL of the Click-iT^®^ reaction cocktail (Thermo Fisher Scientific, Waltham, MA, USA) was added and incubated for 30 min. The cells were washed once with 3% BSA in DPBS, stained with 1 mL of DAPI solution (1 µg/mL in DPBS) for 30 min, and finally washed twice with DPBS. Fluorescence images were acquired using a Nikon Eclipse Ti2 live-cell fluorescence microscope (Tokyo, Japan).

### 4.4. Wound Healing Assay

HDMECs were plated in 6-well culture plates and maintained for 24 h. A linear scratch was then generated at the center of each well using a sterile 1 mL pipette tip to create a uniform wound gap. After gently removing detached cells and debris with DPBS, the cultures were treated with 1 µM DHT, 1 µM MIX, or DPP (0.1, 1, or 5 µM) in fresh medium. The cells were subsequently incubated for an additional 24 h under identical conditions. Phase-contrast images were acquired immediately after scratching (0 h) and after 24 h using a Nikon light microscope (Tokyo, Japan) to assess wound closure.

### 4.5. DCF-DA ROS Assay

Intracellular reactive oxygen species (ROS) levels were evaluated using a Cellular ROS Assay Kit (Abcam, Cambridge, UK). HDMECs were seeded into a confocal dish and incubated for 24 h. The cells were subsequently treated with 1 µM DHT, 1 µM MIX, or 5 µM DPP for 24 h. After treatment, the medium was removed, and the cells were incubated with 10 µM 2′,7′-dichlorofluorescin diacetate (DCF-DA) for 20 min in the dark to measure intracellular ROS levels. The staining solution was then removed, and the cells were rinsed with DPBS. Fluorescence intensity was visualized using a Nikon Eclipse Ti2 live-cell fluorescence microscope (Tokyo, Japan).

### 4.6. Measurement of Mitochondrial Membrane Potential

Mitochondrial membrane potential was assessed using the JC-1 Mitochondrial Membrane Potential Assay Kit (Abcam, Cambridge, UK). HDMECs were seeded into a confocal dish and cultured for 24 h. The cells were subsequently treated with 1 µM DHT, 1 µM MIX, or 5 µM DPP for an additional 24 h. After removing the culture medium, cells were stained with 2 µM JC-1 dye and incubated for 30 min under light-protected conditions. Following staining, the cells were rinsed with DPBS, and fluorescence images were acquired using a Nikon Eclipse Ti2 live-cell fluorescence microscope (Tokyo, Japan).

### 4.7. Measurement of Intramitochondrial ROS

Mitochondrial ROS levels were assessed using MitoSOX™ Red (Invitrogen, Carlsbad, CA, USA). HDMECs were seeded into a confocal dish and cultured for 24 h. The cells were subsequently treated with 1 µM DHT, 1 µM MIX, or 5 µM DPP for an additional 24 h. After removing the culture medium, the cells were incubated with 5 µM MitoSOX™ Red working solution for 10 min under light-protected conditions. Following staining, the cells were rinsed with DPBS and incubated again with MitoLite™ Green FM for another 30 min under the same conditions. Fluorescence images were then acquired using a Nikon Eclipse Ti2 live-cell fluorescence microscope (Tokyo, Japan).

### 4.8. ATP Assay

Mitochondrial ATP production was evaluated using ATP Red™ and MitoLite™ Green FM dyes (AAT Bioquest, Pleasanton, CA, USA). HDMECs were plated in a confocal dish and maintained for 24 h. The cells were treated with 1 µM DHT, 1 µM MIX, or 5 µM DPP for 24 h. The ATP Red™ working solution was then added, and the cells were further incubated for 30 min. The cells were then gently rinsed with DPBS and incubated again with MitoLite™ Green FM for another 30 min under the same conditions. After final washing, fluorescence signals were captured using a Nikon Eclipse Ti2 live-cell fluorescence microscope (Tokyo, Japan).

### 4.9. Western Blot Analysis

HDMECs were plated in 100 mm culture dishes and allowed to adhere for 24 h. The cells were subsequently treated with 1 µM DHT, 1 µM MIX, or DPP at concentrations of 0.1, 1, and 5 µM for 24 h. After treatment, the cells were lysed using RIPA buffer and further centrifuged at 12,000 rpm for 10 min at 4 °C. Protein levels were quantified using a BCA assay kit (Thermo Fisher Scientific, Waltham, MA, USA). Subsequently, 30 µg of protein from each sample was subjected to SDS-polyacrylamide gel electrophoresis and transferred onto polyvinylidene difluoride membranes. The membranes were blocked in 5% skim milk prepared in Tris-buffered saline with 0.1% Tween-20 for 2 h, followed by overnight incubation with primary antibodies directed against p-ERK, ERK, p-JNK, JNK, p-p38, p38, and β-actin (Cell Signaling Technology, Beverly, MA, USA). After washing three times with TBS-T, membranes were exposed to HRP-linked secondary antibodies for 2 h and washed three times before detection. Protein bands were visualized using an ECL detection reagent (Cytiva, Marlborough, MA, USA). Images were captured using the Invitrogen iBright 1500 imaging system (Waltham, MA, USA). Densitometric quantification was performed using ImageJ software (version 1.53e; National Institutes of Health, Bethesda, MD, USA).

### 4.10. Tube Formation Assay

Tube formation was assessed using the Angiogenesis Assay Kit (Cell Biolabs, San Diego, CA, USA). A pre-chilled 96-well plate was coated with 50 μL of Matrigel per well and incubated for 30 min to allow gelation. HDMECs (2.5 × 10^4^ cells/well) were suspended in 150 μL of culture medium and seeded onto the Matrigel-coated wells. After 6 and 24 h of incubation, tube formation was visualized using a Nikon light microscope (Tokyo, Japan).

### 4.11. Statistical Analysis

All data are presented as mean ± standard deviation (SD). Error bars indicate SD values obtained from independent experiments. Statistical comparisons among groups were conducted using one-way analysis of variance (ANOVA) followed by appropriate post hoc tests. Statistical analyses were conducted with GraphPad Prism software (version 8.01, San Diego, CA, USA).

## Data Availability

The data presented in this study are available on request from the corresponding author.
